# Inclusive Leadership, Psychological Safety, AI Readiness, and Nurses’ AI Literacy: A Longitudinal Study

**DOI:** 10.1155/jonm/9935514

**Published:** 2026-08-19

**Authors:** Yongkang Fu, Qixu Zhou, Guiyan Shi, Chaoran Chen, Enshe Jiang, Jiangong Ma

**Affiliations:** ^1^ Department of Neurosurgery, The First Affiliated Hospital of Henan University, Henan University, Kaifeng 475004, China, henu.edu.cn; ^2^ Institute of Nursing and Health, School of Nursing and Health, Henan University, Kaifeng 475004, China, henu.edu.cn

**Keywords:** AI literacy, AI readiness, inclusive leadership, nurses, psychological safety

## Abstract

**Aim:**

To investigate the relationship between inclusive leadership and artificial intelligence (AI) literacy among nurses, with a specific emphasis on whether psychological safety and AI readiness mediate this relationship sequentially.

**Methods:**

A longitudinal study was conducted between September 2025 and March 2026 with the use of a three‐wave survey on 1334 clinical nurses. The selected hospitals were stratified by cluster random sampling at 10 tertiary hospitals in China. In T1, the level of inclusive leadership was measured, and in T2, the psychological safety as well as AI readiness was also tested. At T3, the AI literacy was assessed based on the tools that had been validated before. SPSS 25.0 and PROCESS 4.1 were used to test mediating effects.

**Results:**

All the important factors were positively correlated. The study also did not have any direct correlation between AI literacy and inclusive leadership. Nevertheless, it was passed through a number of indirect ways. The psychological safety (indirect effect = 0.258, 95% confidence interval [CI] [0.205, 0.315]) and the AI readiness (indirect effect = 0.082, 95% CI [0.051, 0.115]) were significant mediators in the relationship. Also, a sequential path was indicated by the data: as more inclusive leaders were observed to be, the more psychologically safe they became, which would then result in increased AI readiness, and finally, this resulted in AI literacy among nurses (indirect effect = 0.049, 95% CI [0.030, 0.070]).

**Conclusions:**

The psychological safety and the AI readiness are two of the factors that can be used to explain why inclusive leadership is associated with the AI literacy among nurses. Our findings reveal that our study does not directly affect the AI literacy, but it affects how the nurse feels about the interpersonal relationship in the workplace as well as her ability to cope with the demands of the AI technology. That is, the impact of the inclusive leadership on the AI literacy of the nurses will be clearer when the nurses are psychologically safe and prepared to use the AI.

**Implications for Nursing Management:**

Our results can be used to determine that leadership behavior is a very important part of the organization, and it should provide open communication and learning in the workplace. The development of AI literacy will occur when the managers in the nursing field are able to establish psychological safety and enable nurses to have an increased level of confidence in the use of new technologies. These outcomes can also be enhanced through organizational initiatives like the education and skill building programs on AI.

## 1. Introduction

With the rapid iteration and widespread application of artificial intelligence (AI), the healthcare sector is undergoing a profound digital transformation [1]. The use of AI to make intelligent diagnostic systems, clinical decision making tools, and smart nursing systems are some of the innovations that have changed the way healthcare services can be performed and how the professionals perform their work [2, 3]. It has made the nursing practice more complicated. This is because this situation makes it more challenging on the part of the nurses to understand technology and adapt to it and interact with AI [4]. There is a need to apply AI at high intensity and high risk conditions, which is an issue that now needs to be addressed as it affects the quality of care and safety of patients. That is why the development of the literacy of the nurses towards the application of AI has become urgent and practical [5, 6].

Nurses are the largest professional category in healthcare and, as a result of their profession, they are the first to introduce AI technologies into their work. In fact, the nurses will be able to use AI tools to some extent because they can comprehend and use them correctly [7]. Intelligent healthcare system is implemented by the nurses. Being the main users of various AI‐based technology, they are capable of influencing the results of such implementation of AI systems in the healthcare industry [8]. The nursing practice is a complex, emotional, and high responsibility task. Such features can cause the nurse to have issues with adaptation and cognitive overload when it comes to new technologies [9]. As the direct implementers of AI applications and key agents in the transformation toward healthcare intelligence, nursing staff are required not only to master relevant technical competencies but also to cope with adaptive pressures arising from role transitions and the restructuring of work practices. Therefore, examining the developmental mechanisms of AI literacy from the perspective of the nursing workforce not only contributes to a deeper theoretical understanding of the pathways through which nurses’ AI literacy is formed but also provides important practical guidance for healthcare institutions in formulating targeted management and training strategies.

AI literacy is a set of skills, which can be used to analyze AI technologies critically, and collaborate with AI in the online environment, at home, and at work. This definition highlights the technical knowledge, moral contemplation, and the practical implementation of the idea as opposed to the development of AI [10]. The increased AI literacy has been associated with the trust in intelligent systems in the nursing practice and more acceptance of the AI‐based technology in nursing. These abilities could also enhance better science based and safer decision‐making in the nursing profession and enhance the quality of care [11]. Existing research has started to identify factors associated with AI literacy, such as technological attitudes, digital competence, and organizational support [12]. However, the current evidence has several limitations. First, most studies focus on students or employees in general occupational settings; nurses have received relatively little attention [13]. Second, methodological issues exist. Cross‐sectional designs dominate the literature, which limits our understanding of how AI literacy develops over time [14]. Third, although organizational conditions are often mentioned as influential, the specific pathways through which leadership affects nurses’ AI literacy remain unclear. In particular, how individual psychological states and readiness‐related capabilities mediate leadership effects has not been fully explored [15].

To address these gaps, this study focuses on inclusive leadership as a key organizational antecedent of nurses’ AI literacy. Psychological safety and AI readiness are proposed as sequential mediators between leadership behaviors and literacy development. Using a longitudinal design, we examine whether inclusive leadership enhances psychological safety and AI readiness, and whether these factors in turn promote AI literacy among nurses. The findings are expected to extend theoretical understanding while offering practical guidance for nursing management and workforce development in an increasingly AI‐enabled healthcare environment.

## 2. Background

### 2.1. Inclusive Leadership and Nurses’ AI Literacy

Leadership is one of the organizational variables that are essential in healthcare facilities where intelligent technologies have been introduced to a higher level and which has been used to influence the process of nurses adapting to new technology and acquiring the skills [16]. In this general context, the concept of inclusive leadership has become more and more popular among the scholars of both the field of organizational behavior and nursing management [17]. It is usually referred to as an open and fair leadership style with positive relations. By such actions, leaders will establish a workplace that recognizes and respects diversity in the team [18]. This form of leadership also pays attention to the development of employees, as well as their ability to achieve professional value, which is why it can be used in complex and unpredictable work situations [19]. According to the social information processing theory, the people can read signals provided by their surroundings at the organization and the conduct of the leader, and they can deduce what kind of behaviors and skills should be promoted and rewarded [20]. Nurses who perceive openness to new technologies, patience toward learning, and acceptance of errors would tend to view technology in a more positive light and feel more inclined to learn [21, 22]. The interaction with AI‐related tools under such circumstances will be active, not passive. This may eventually result in long‐term efforts to make sense of the use of AI and its application in practice. Meanwhile, the inclusive leadership is able to build a friendly environment in the organization, which contributes to the psychological safety of the nurses and the identification of the nurses with the organization [23]. This atmosphere reduces the risk perception of the interpersonal relationship between the individuals attempting to adopt the new technologies and thus makes them more ready to take on the risks associated with the adoption of the new technologies. As the nurse continues to interact with the AI systems in such an atmosphere, she or he can become competent and confident over time, which will enable her or him to continue working with AI literacy through practice. Previous studies on the subject matter have already indicated the importance of the idea of inclusive leadership in digital workplaces. It was found that it helps the employees to develop learning practices, innovative behaviors, and adaptability to the technological changes and even indirectly improve individual performance based on psychological empowerment, engagement in learning, and self‐efficacy [24]. Accordingly, this study proposes Hypothesis 1: Inclusive leadership positively influences nurses’ AI literacy.

### 2.2. The Mediating Role of Psychological Safety

Psychological safety is a feeling of psychological security and comfort in the workplace where the employees are free to voice their views, make mistakes, and take risks without being judged or punished on the basis of the image, position, reputation, and career [25]. Psychological safety has been considered one of the key factors that have helped nurses in the nursing setting where there is a high level of technological progress and professional specialization in the field [26]. According to the theory of psychological safety, inclusive leadership can be defined as an antecedent condition of the psychological safety because it provides the working environment with the atmosphere of respect towards individual diversity, motivation of participation, and tolerance of the mistakes and failures [27]. These circumstances decrease the risk of interpersonal relations and allow the learning behaviors. The association between more psychological safety in healthcare and nursing environments and the increased frequency of reflection and adaptability to new technology among nurses has also been found [28]. AI literacy is not about getting the knowledge. It is based on trial and error process of practice that involves a lot of uncertainty and even potential failure [29]. This is how the situation arises where psychological safety is a facilitative factor. A nurse who feels that she or he is psychologically safe will tend to be more open and active in the area of learning and applying AI technologies than those who are hesitant. Such inclination gives a necessary mental preparation in order to keep interacting with the AI technologies and eventually acquire the skills of AI literacy [30]. On the other hand, the absence of psychological safety can limit the ability of nurses to be proactive and persistent in acquiring and implementing AI systems [31]. Therefore, this study proposes Hypothesis 2: Psychological safety mediates the relationship between inclusive leadership and nurses’ AI literacy.

### 2.3. The Mediating Role of AI Readiness

The AI preparedness in the nursing field is the level of readiness among nurses to apply the medical AI systems, which are combined with their professional knowledge and skills in delivering services of prevention, diagnosis, treatment, and rehabilitation [32]. The AI readiness has been a crucial psychological and capability mechanism between the external technological environments and the individual responses to technology; it has also been considered as an important requirement to adapt successfully to the intelligent transformation and implement the concept of empowerment through the use of technology in various spheres including healthcare, education, and organizational management [33]. It has been theorized that leaders are role models in the social information processing theory. Their open‐minded attitude toward new technologies, the positive interaction, and the learning behavior of the nurse can send the normative message about the AI perception and the desire to learn something related to AI [13, 34]. The literature has shown that inclusive leadership by creating an environment where the nurses feel at ease and comfortable with diversity and openness to change and errors will reduce anxiety and uncertainty among the nurses in relation to changes brought about by the use of technology and hence increase confidence and willingness to work with AI systems [35]. AI preparedness is one of the proximal factors of the process of AI literacy formation of the nurses [36]. Nurses who are more ready to AI do many things. They have learned how AI works and what it does. They also believe in technology. Also, they employ AI in clinical settings. This leads them to improve on their AI literacy [37, 38]. In the case when there is no readiness, this situation will be different. There may be some resistance or fear of adopting technology. Or perhaps it is not enough to get good results even though there are resources to provide [39]. Accordingly, this study proposes Hypothesis 3: AI readiness mediates the relationship between inclusive leadership and nurses’ AI literacy.

### 2.4. The Chain Mediating Role of Psychological Safety and AI Readiness

The theory of social information processing suggests that people will be able to modify their thinking, feelings, and actions depending on the social data provided in the working conditions. In the nursing environment, nurses are also able to perceive the safety of the surrounding, especially the psychological safety, by the way of perceiving cues of being supported, included, and encouraged in the leadership behaviors [40, 41]. The degree of psychological safety can have an impact on the willingness and readiness of nurses to learn AI technologies and thus, it is a factor that can also impact the level of AI literacy. Research indicates that the greater the sense of psychological safety among nurses, the more they are likely to adopt new technology, as well as participate in training and learning processes, which, in its turn, can make them ready to work with AI and practice the use of AI [42]. On the other hand, when there is not enough psychological security, then this may decrease the motivation to learn and the eagerness to implement the AI and hence limit the growth of the readiness and skills of the AI [43] Accordingly, this study proposes Hypothesis 4: Psychological safety and AI readiness sequentially mediate the relationship between inclusive leadership and nurses’ AI literacy.

**FIGURE 1:**
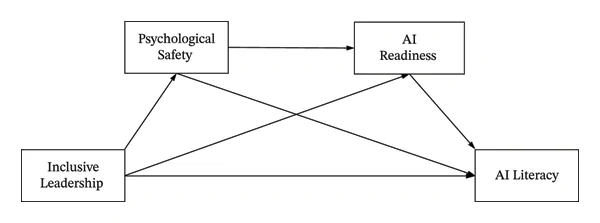
Theoretical framework.

In conclusion, based on the four hypotheses mentioned above, this study constructs a chain mediation model combining inclusive leadership, psychological safety, AI readiness, and nurses’ AI literacy to investigate the mechanisms by which inclusive leadership affects nurses’ AI literacy and the serial mediating roles of psychological safety and AI readiness (Figure 1).

## 3. Methods

### 3.1. Research Design

The research design of the study was longitudinal questionnaire–based survey. It looked at the relationships between inclusive leadership, psychological safety, AI readiness, and nurses’ AI literacy. This design recorded data with time and enabled the investigation of possible causal relations. The information was gathered by means of self‐reported questionnaires in three moments (T1, T2, and T3). A chain mediation model was then developed to examine the mediating effects of psychological safety and AI readiness. The research adhered to the STROBE guidelines in order to make it transparent and of high reporting quality.

### 3.2. Participants

The recruitment of nurses was done in 10 tertiary general hospitals in China. The departments were the main sampling units within each hospital. A stratified cluster sampling method was applied to randomly select nurses across various clinical departments. This technique increased the representativeness and heterogeneity of the sample. Altogether, 1500 questionnaires were distributed and finally 1334 valid responses were received.

The selection criteria were as follows: (1) registered clinical nurses, (2) nurses who provided informed consent and voluntarily participated in the survey, and (3) nurses who had worked in their current hospital for at least 1 year. The exclusion criteria included (1) nurses not directly involved in patient care and nurses undergoing advanced training and (2) questionnaires with substantial missing data, omissions, or invalid responses. Before the study started, approval was obtained from hospital administrators, and informed consent was secured from all participants. These procedures ensured the ethical integrity of the research.

### 3.3. Data Collection

Data were collected at three time points: September 2025 (T1), December 2025 (T2), and March 2026 (T3). At each wave, participants completed questionnaires covering demographic information, inclusive leadership, psychological safety, AI readiness, and AI literacy.

To improve traceability and data rigor, each participant received a unique anonymous identification number (ID) at T1. The same ID was used at T2 and T3 to ensure accurate matching across time points. At T1, 1500 questionnaires were distributed, and 1436 valid responses were collected. Three months after T1, the second wave (T2) was conducted. After excluding cases with missing data, unmatched IDs, and patterned responses, 1389 valid questionnaires were retained. Three months after T2, the third wave (T3) was conducted using the same procedures and exclusion criteria. This yielded a final sample of 1334 valid questionnaires, with an effective response rate of 88.93%.

The cross time ID matching was confirmed during the entire data collection. This made sure that the information of the same individuals would be matched between T1, T2, and T3. The quality control process of these systematic procedures was also a factor in making sure that the study data are reliable and scientifically sound.

### 3.4. Measures

#### 3.4.1. Inclusive Leadership

Inclusive leadership was measured using the Inclusive Leadership Scale developed by Carmeli et al. [44] and sinicized by Wang [45]. The scale consists of 9 items across three dimensions: openness, availability, and accessibility. Responses were rated on a 5‐point Likert scale ranging from 1 (“*strongly disagree*”) to 5 (“*strongly agree*”), with higher scores indicating higher levels of inclusive leadership. The original Cronbach’s *α* of the scale was 0.94. In recent research, the Cronbach’s *α* was 0.829, and confirmatory factor analysis (CFA) indicated a good model fit: CMIN/DF = 1.899, RMSEA = 0.019, CFI = 0.997, TLI = 0.996, and GFI = 0.988 [46]. In the present study, the scale demonstrated good reliability and validity (Cronbach’s *α* = 0.935; KMO = 0.943).

#### 3.4.2. Psychological Safety

Psychological safety was assessed using the Psychological Safety Scale developed by Edmondson [47] and translated into Chinese by Liu [48]. This unidimensional scale contains 7 items rated on a 7‐point Likert scale from 1 (“*strongly disagree*”) to 7 (“*strongly agree*”). Higher scores indicate higher levels of psychological safety. The original Cronbach’s *α* was 0.82. In recent studies, the Cronbach’s *α* reached 0.94, with CFA results indicating a good fit (CMIN/DF = 1.871, RMSEA = 0.042, CFI = 0.989, TLI = 0.984, and GFI = 0.986). In this study, the Cronbach’s *α* coefficient was 0.882, and the KMO value was 0.874.

#### 3.4.3. Medical AI Readiness

AI readiness was measured using the Medical Artificial Intelligence Readiness Scale developed by Karaca et al.[32] and translated and adapted into Chinese by Li Jizhang et al. [49]. The scale consists of 20 items covering four dimensions: cognition, capability, vision, and ethics. Items were rated on a 5‐point Likert scale ranging from 1 (“*strongly disagree*”) to 5 (“*strongly agree*”), with total scores ranging from 20 to 100. Higher scores indicate higher levels of AI readiness. The original Cronbach’s *α* of the scale was 0.87. In recent research, the Cronbach’s *α* was 0.778, and CFA results demonstrated a good fit (CMIN/DF = 1.127, RMSEA = 0.018, CFI = 0.946, TLI = 0.955, and GFI = 0.957). In the present study, the scale demonstrated excellent internal consistency (Cronbach’s *α* = 0.966; KMO = 0.964).

#### 3.4.4. AI Literacy

Nurses’ AI literacy was measured using the Clinical Nurses’ Artificial Intelligence Literacy Scale developed by Wang et al. [50] and sinicized by Kong [51]. The scale consists of 12 items divided into four dimensions: awareness, use, evaluation, and ethics. Responses are rated on a 7‐point Likert scale, where 1 means “*strongly disagree*” and 7 means “*strongly agree*.” Total scores range from 12 to 84. Higher scores reflect higher levels of AI literacy. The original Cronbach’s *α* of the scale was 0.83. In recent studies, the Cronbach’s *α* was 0.829, and CFA results indicated a good fit (CMIN/DF = 1.034, RMSEA = 0.01, CFI = 0.99, TLI = 0.99, and GFI = 0.98). In this study, the scale showed good reliability and construct validity (Cronbach’s *α* = 0.899; KMO = 0.940).

### 3.5. Data Analysis

Data were analyzed using IBM SPSS Statistics 25.0 and PROCESS Version 4.1. First, Harman’s single‐factor test was conducted to assess common method bias (CMB), and frequencies and percentages were used to describe participants’ demographic characteristics. Second, Pearson’s correlation analysis was performed to examine the relationships among inclusive leadership, psychological safety, AI readiness, and AI literacy. When variables did not conform to a normal distribution, Spearman’s correlation analysis was applied.

Finally, the chain mediating effects of psychological safety and AI readiness in the relationship between inclusive leadership and nurses’ AI literacy were tested using Hayes’ PROCESS macro (Model 6). A bootstrap procedure with 5000 resamples was used to estimate bias‐corrected 95% confidence intervals (CIs). All statistical tests were two‐tailed, and *p* < 0.05 was considered statistically significant.

### 3.6. Ethical Considerations

This study was conducted as an anonymous survey and did not involve unethical behavior or human clinical experiments nor did it cause physical or psychological harm to participants. The study was conducted in accordance with the Declaration of Helsinki and was reviewed and approved by the Ethics Committee of the Biological Sciences Research Group at Henan University (Approval No. HUSOM2025‐1017). Prior to questionnaire distribution, each participant was fully informed of the study’s purpose, procedures, voluntary nature, and confidentiality measures, and written informed consent was obtained. In addition, permission was secured from hospital administrators and nursing departments to conduct the survey within their institutions. Participation was entirely voluntary, and all data were treated with strict confidentiality.

## 4. Results

### 4.1. CMB Test

Because all variables were obtained through self‐reported measures, CMB was assessed using Harman’s single‐factor test. The results showed that the first factor accounted for 43.24% of the total variance, which is below the 50% threshold. Therefore, no serious CMB was detected in this study [52].

### 4.2. The Demographic Characteristics of the Participants

A total of 1334 nurses participated in this study, including 69 males (5.2%) and 1265 females (94.8%). Most participants held a bachelor’s degree or higher (92.4%), and the majority were married (75.1%). Most nurses had between 2 and 19 years of working experience (82.6%). In addition, 58.6% of the participants were employed under personnel agency contracts. Detailed demographic information is presented in Table 1.

**TABLE 1:** Demographic characteristics of the participants (*N* = 1334).

Demographics	*N* (%)
Gender	
Male	69 (5.2)
Female	1265 (94.8)
Age (year)	
< 35	670 (50.2)
≥ 35	664 (49.8)
Marital status	
Single	301 (22.6)
Married	1002 (75.1)
Others	31 (2.3)
Education	
≤ Associate’s degree	102 (7.6)
Bachelor’s degree	970 (72.7)
≥ Master’s degree	262 (19.6)
Labor and personnel relations	
Formal establishment	218 (16.3)
Personnel agency	782 (58.6)
Contract system	334 (25.0)
Years of working	
< 2	68 (5.1)
2–9	521 (39.1)
10–19	581 (43.5)
≥ 20	164 (12.3)
Monthly income (RMB)	
< 6000	234 (26.7)
6000–10,000	673 (50.4)
10,001–15,000	275 (20.6)
> 15,000	152 (11.3)

*Note:* Others: divorced or widowed.

### 4.3. Correlation Analysis and Significance Testing

The means (M), standard deviations (SDs), and Pearson’s correlation coefficients for all study variables are presented in Table 2. As inclusive leadership, psychological safety, AI readiness, and nurses’ AI literacy across the three time points were all normally distributed, Pearson’s correlation analysis was conducted to examine the relationships among these variables. The results showed that inclusive leadership at T1 was positively correlated with psychological safety at T2 (*r* = 0.671, *p* < 0.01), AI readiness at T2 (*r* = 0.659, *p* < 0.01), and AI literacy at T3 (*r* = 0.413, *p* < 0.01). In addition, psychological safety at T2 (*r* = 0.528, *p* < 0.01) and AI readiness at T2 (*r* = 0.461, *p* < 0.01) were both positively correlated with AI literacy at T3. Finally, a significant positive correlation was also observed between psychological safety and AI readiness at T2 (*r* = 0.644, *p* < 0.01).

**TABLE 2:** Descriptive statistics and correlations among the study variables (*N* = 1334).

	Mean	Standard deviation	1	2	3	4
1. IL	36.48	5.80	1			
2. PS	28.59	3.98	0.671^∗∗^	1		
3. AI‐R	71.39	12.11	0.659^∗∗^	0.644^∗∗^	1	
4. AI‐L	58.97	8.45	0.413^∗∗^	0.528^∗∗^	0.461^∗∗^	1

Abbreviations: AI‐L, AI Literacy; AI‐R, AI Readiness; IL, Inclusive Leadership; PS, Psychological Safety.

^∗∗^ *p* < 0.01 (two‐tailed).

### 4.4. Testing the Chain Mediation Model

**FIGURE 2:**
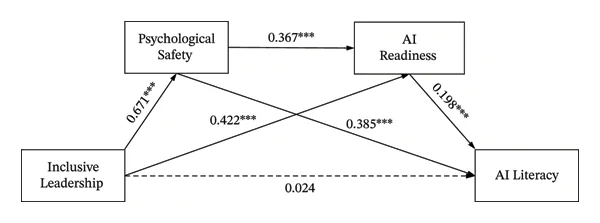
A longitudinal mediating model of Inclusive Leadership, Psychological Safety, AI Readiness, and AI Literacy.

We tested the chain mediation model using the SPSS PROCESS macro (Model 6). The results indicated that inclusive leadership positively predicted AI literacy (*β* = 0.413, *p* < 0.001; Model 1). Inclusive leadership also positively predicted psychological safety (*β* = 0.671, *p* < 0.001; Model 2). In addition, inclusive leadership positively predicted AI readiness (*β* = 0.413, *p* < 0.001; Model 3), and psychological safety positively predicted AI readiness (*β* = 0.367, *p* < 0.001; Model 3). When inclusive leadership, psychological safety, and AI readiness were simultaneously entered into the regression equation (Model 4), psychological safety positively predicted AI literacy (*β* = 0.385, *p* < 0.001), and AI readiness also positively predicted AI literacy (*β* = 0.198, *p* < 0.001). Detailed results are presented in Table 3 and Figure 2.

**TABLE 3:** Testing the chain mediation model (*N* = 1334).

Predictive variable	Model 1 (dependent variable: SB)		Model 2 (dependent variable: PS)		Model 3 (dependent variable: AI‐R)		Model 4 (dependent variable: AI‐L)	
	*β*	*t*	*β*	*t*	*β*	*t*	*β*	*t*
EL	0.413	16.528^∗∗∗^	0.671	33.064^∗∗∗^	0.413	15.918^∗∗∗^	0.024	0.706
PS					0.367	14.150^∗∗∗^	0.385	11.619^∗∗∗^
AI‐R							0.198	6.074^∗∗∗^
*R* ^2^	0.170		0.451		0.508		0.304	
*F*	273.186^∗∗∗^		1093.194^∗∗∗^		688.246^∗∗∗^		193.934^∗∗∗^	

Abbreviations: AI‐L, AI Literacy; AI‐R, AI Readiness; IL, Inclusive Leadership; PS, Psychological Safety.

^∗∗∗^ *p* < 0.001 (two‐tailed).

Finally, the mediating effects were further examined using the bias‐corrected percentile bootstrap method with 5000 resamples. Mediation effects were considered statistically significant when the 95% CIs did not include zero. The results indicated that the direct effect of inclusive leadership on AI literacy was not significant, whereas all mediating effects reached statistical significance. Table 4 shows that inclusive leadership affected AI literacy through the following pathways:

a. Inclusive Leadership⟶ Psychological Safety ⟶  AI Literacy (indirect effect: 0.258, 95% CI: [0.205, 0.315]).
b. Inclusive Leadership ⟶  AI Readiness ⟶  AI Literacy (indirect effect: 0.082, 95% CI: [0.051, 0.115]).
c. Inclusive Leadership ⟶  Psychological Safety ⟶  AI Readiness ⟶ AI Literacy (indirect effect: 0.049, 95% CI: [0.030, 0.070]).

**TABLE 4:** Bootstrap analysis of the mediating model (*N* = 1334).

Effects	Paths	Effect	SE	95% CI	Percentage
Total effect	T1IL ⟶ T3AI‐L	0.413	0.025	0.364–0.462	100%

Direct effect	T1IL ⟶ T3AI‐L	0.024	0.034	−0.042–0.090	5.81%

Indirect effect	T1IL ⟶ T2PS ⟶ T3AI‐L	0.258	0.028	0.205–0.315	62.47%
	T1IL ⟶ T2AI‐R ⟶ T3AI‐L	0.082	0.017	0.051–0.115	19.86%
	T1IL ⟶ T2PS ⟶ T2AI‐R ⟶ T3AI‐L	0.049	0.010	0.030–0.070	11.86%

Total indirect effect	Total indirect effect	0.389	0.031	0.328–0.452	94.19%

Abbreviations: AI‐L, AI Literacy; AI‐R, AI Readiness; IL, Inclusive Leadership; PS, Psychological Safety.

## 5. Discussion

### 5.1. Association of Inclusive Leadership With AI Literacy of Nurses

The longitudinal research design of the study developed a mediation model in which the impact of inclusive leadership on AI literacy among nurses was examined, as well as the mediating effect of psychological safety and AI readiness. It was found that there was a positive relationship between the two variables, i.e., inclusive leadership and the AI literacy of nurses. But once psychological safety and AI readiness were added to the mediators, it did not have significant effects on AI literacy with regard to inclusive leadership directly, but this is inconsistent with Hypothesis 1. Our conclusion is that the AI literacy of the nurses is linked to the concept of an inclusive leader. Nevertheless, its impact does not occur by way of a straight path. Rather, it is based on the more complicated mediation process. The above findings are in line with the earlier studies of technology capability building. This has demonstrated that one leadership behavior cannot be used to shape high order technical literacy. It is usually a product of a number of psychological and capability processes [53, 54]. There might be two reasons why the direct impact is insignificant: first, the higher‐order ability of AI literacy is accumulated at the level of psychological cognition and readiness to apply the abilities, and this is done with the support of the context and individual conditions [55, 56]. Second, the impact of the inclusive leadership can also be stronger in creating positive work settings and developmental environment than the enhancement of complex capabilities [26]. In addition, the nonsignificant direct longitudinal effect of inclusive leadership on nurses’ AI literacy may be attributable to the relatively long developmental cycle of AI literacy, given that this study employed three measurement waves with 3‐month intervals. Although inclusive leadership can improve employees’ work experiences and psychological states within a relatively short period, AI literacy, as a higher‐order capability, typically requires extended periods of knowledge accumulation and practical application. Therefore, within the time frame covered by this study, the influence of inclusive leadership may first manifest in enhanced psychological safety and AI readiness, rather than being directly translated into significant improvements in AI literacy. Thus, inclusive leadership is not a stand‐alone practice of increasing the AI literacy of the managers. Instead, they must integrate their leadership practices and supportive systems, including training resources and organizational structures, to increase the AI literacy of the nurses. Specifically, the role of the managers is to monitor the activities of the leaders and how they affect the working environment of the workers and the mental health of the nurses. Such actions provide the necessary prerequisites to the emergence of the competences associated with AI. Meanwhile, the inclusion of inclusive leadership should be combined with other approaches such as the training tools, learning opportunities, and institutional arrangements. It may lead to the creation of synergistic effects and the AI literacy of the nurses in a systematic manner and in a sustainable fashion.

### 5.2. The Mediating Role of Psychological Safety

The main result of the current research is that inclusive leadership was an important mediator in the relationship between nurses and their AI literacy, and psychological safety has been found to be one of the mediators, which proves the Hypothesis 2. In other words, when a leader is an inclusive leader, it will help to create psychological security in the nurses who are then likely to have positive effects on their AI knowledge. These results not only prove the significance of the role of psychological safety but also prove the long‐term effect of psychological safety. To be more precise, Time 1 nurses with high levels of perceived inclusive leadership were associated with increased rates of psychological safety at Time 2 and these rates predicted greater rates of AI literacy at Time 3. This implies that the psychological safety does not occur as a reaction to the behavior of the leader and instead becomes a vital resource of the mind that can be used over time to facilitate further learning and development of capabilities. There is already some evidence to this conclusion. The employees become more psychologically safe because of the presence of inclusive leadership, and this is highly correlated with the willingness of nurses to learn and even implement new technologies [57, 58]. According to the theory of psychological safety, people tend to take risks more readily when they believe that no harm would come out of them speaking up or doing something new [59, 60]. It is a risky job. The introduction of AI brings about additional unpredictability: the technology itself is unfamiliar, the roles are vague, the possibility of mistakes. Nurses are prone to psychological anxiety in such situations [61, 62]. Therefore, the psychological safety should also be considered as the basis of AI [63]. Inclusive leadership can be established by allowing the process of questioning and trial‐and‐error. Openness, accessibility, and respect to various opinions among leaders make the cognitive defensiveness and emotional load less severe. As a consequence, nurses are less hesitant to recognize the weaknesses and seek assistance themselves and keep learning [31], which eventually leads to the gradual growth of mature and reasonable AI literacy in practice [64]. Consequently, it is necessary for nursing managers to understand that the psychological safety of the team cannot be regarded as a desirable condition, but it is rather the condition, without which it is impossible to improve the level of AI literacy of nurses. To illustrate, organizations may use institutional structure, communication systems, training processes, etc. to strengthen the feedbacks of the question, experiment, and reflection. Similarly, the training of nurses in AI‐related courses must involve the creation of a psychologically safe environment where the nurse is able to convert psychological safety into motivation to pursue the goal of achieving AI literacy.

### 5.3. The Mediating Role of AI Readiness

Another important finding is that AI readiness significantly mediates the relationship between inclusive leadership and nurses’ AI literacy, providing empirical support for Hypothesis 3. This finding aligns with prior research on technology readiness and technology adoption, which has consistently shown that individuals’ readiness for new technologies is a critical antecedent of digital skill development and technology use and that organizational support and leadership behaviors can effectively enhance healthcare professionals’ readiness for technological change [65, 66]. Nevertheless, the current literature has mostly considered AI readiness to be an immediate antecedent of technology adoption or behavior of short term usage, instead of focusing on the importance of the critical intermediary role of AI readiness in the overall development of professional capabilities over a longer period. On the contrary, the study currently under discussion broadens the dependent variable, AI literacy, to the higher‐order general ability and confirms its mediational effect between the leadership action and the capability growth using the longitudinal research design, which clarifies its place in the system of the capability building. From a temporal sequencing perspective, the effect of inclusive leadership on AI readiness exhibits a certain time‐lagged characteristic. Specifically, after perceiving leadership support, nurses do not immediately demonstrate improvements in AI literacy; rather, they gradually develop understanding, confidence, and willingness to apply AI technologies, thereby entering a “state of readiness.” This readiness is subsequently translated into the development of AI literacy in later stages, indicating that AI readiness serves as a critical transitional mechanism linking organizational support and capability formation. In terms of mechanics, the concept of AI readiness is characterized by not only the knowledge of the individuals about the nature of AI technology and the confidence of learning but also the adjustment of the psyche and the intention to act in response to the changes in technology [67]. Inclusive leadership could contribute to the creation of the feeling of control and the self‐efficacy of learning among the nurses during the adaptation of the technology because it can provide them with resources and promote continuous learning and acknowledge their efforts [68, 69]. Additionally, the greater the AI readiness of nurses, the more actively they understand the features and shortcomings of the AI systems and how to improve the understanding of the system through the process of practicing the system, and this is why the AI readiness of the nurses leads to the formation of the AI literacy [70, 71]. Moreover, the readiness to adopt AI is more directly connected with the presence of conditions in individuals, which would allow them to convert the intention of learning into the real competencies than the psychological safety, which mainly serves as the means of reducing the psychological distress of individuals when confronted with uncertainty. Put differently, the willingness to try is based on the psychological safety whereas the AI readiness is based on the ability to master. Lack of adequate readiness even within a safe and encouraging atmosphere does not mean that people will necessarily be able to learn in a way that will make them better at what they do. Thus, AI preparedness is a bridge that cannot be ignored. Based on the abovementioned evidence‐based data, the nursing organizations and healthcare facilities ought to consider the development of AI readiness among the nurses as one of the intermediate steps in the process of promoting the AI literacy among the nurses in the clinical setting. With the help of systematic training, tiered learning material appropriate to the position of the nurse, and well‐planned step‐by‐step practical activities, frontline managers in the field of nursing can effectively introduce the conceptual understanding, confidence in practice, and active desire to learn about the new technology of AI. These positive actions will not only enhance the achievement of the AI readiness but also ensure the future success of achieving the full‐scale AI literacy of the nurses.

### 5.4. The Serial Mediating Roles of Psychological Safety and AI Readiness

Lastly, the paper has established that psychological safety and AI preparedness of inclusive leadership to AI literacy in nurses had a significant serial mediation effect, which is in favor of Hypothesis 4. The results of these findings further explain the dynamic mechanism of how the process of the development of the AI literacy of nurses occurs. In particular, when it comes to inclusive leadership, there is a stage where the nurse becomes more psychologically safe, at an early age; then, as the level of psychological safety increases, confidence in learning and applying AI technologies increases, and this leads to greater levels of readiness to use AI technologies; finally, as the levels of readiness to use AI technologies increase, they become more ready to develop AI literacy. This means that the technological readiness of the nurses does not occur immediately, but the process of its occurrence involves a gradual process of supportive leadership environment–accumulation of psychological resources–technological readiness–capability formation. Although both the role of psychological safety in the behavior of learning and the growth of capabilities have been discussed in literature [72] and the impact of technology readiness on digital skills [73, 74], there are very few works where the authors have tried to combine the variables in one model by using longitudinal design. This study is different from previous cross‐sectional studies since it has confirmed the roles played by psychological safety and AI readiness and also explained their order of importance in time. These two outcomes of the serial mediation test provided by the current research give a much more consistent and well‐rounded explanation framework, indicating that the psychological safety is a more basic psychological precondition, which will allow people to move toward a state of being ready to work with technology [75, 76], whereas AI readiness is a closer capability condition, directly leading to the emergence of AI literacy among nurses. It can be theoretically explained that this sequence of events is a plausible one: the competencies of the nurses in relation to AI change step by step. Psychological security makes people feel less riskier in terms of uncertainty [77] and allows the nurses to admit their shortcomings, ask questions, and explore the opportunities offered by the AI technology [78, 79]. After the establishment of the psychological safety, the nurse tends to learn about the AI actively and gradually develops the understanding, confidence and intention to apply it ([80], [81]). Such conditions of readiness are then followed by the need to achieve AI literacy [40]. A stepwise intervention approach would make sense in the case of nursing organizations and managers. First, the inclusive leadership should be employed to create a positive working atmosphere and provide the required psychological conditions of the AI learning. Second, AI readiness can be achieved through systematic training and practical assistance, and the assessment of capabilities to achieve AI literacy. As a teacher in the field of education, the nurse educators must consider incorporating real‐life clinical situations into the simulation and case‐based teaching and provide continuous technical assistance and provision of materials. The curriculum needs to be developed with the help of psychological support and motivation to learn. The consequence of this is that the AI education system will be more sensitive to the context and sustainable because it will meet the demands of the nurses.

## 6. Significance to Practice

The study reveals that the inclusive leadership is a psychological safety and AI readiness, not directly. Therefore, to make nurses more literate in AI, managers should concentrate on these two intermediate stages. The first step will be the psychological safety. Nurses are less likely to be anxious about new technologies because of leaders who are open‐minded, allow other opinions, and provide constructive responses to any questions. The outcome? The nurses are able to learn, accept, and even adapt to the use of AI tools. That means it is not just the work environment which is nice to have but it is mandatory. There is also the second piece of AI readiness. Psychological safety has been established; therefore, the confidence and know how to do things should be built by the nurse. This can be achieved through staged training, actual situations, and simulation. It is important to plan gradual steps: at the beginning, it is simple and then gradually complex. Only when the nurses are prepared to put into practice what they were taught will the support offered by an organization result in real competence. The serial path provides the practical guide. Since there is the ability to prepare AI as well as the ability to read AI, the manager can adjust his or her approach depending on the starting point of the department. Other units may require further development of their safety (e.g., ask questions without being blamed); others may need to be trained. The main thing is to create AI literacy based on the principle of psychological security—this is how the organizations can develop AI literacy and overcome the problem of fast change in AI.

## 7. Limitations

Despite its contributions, this study has some limitations. Self‐report bias: All data were derived from nurses self‐reports, which is prone to bias. Future studies might employ mixed methods: experimental designs, peer ratings, or qualitative interviews. Sample restricted to large hospitals: We recruited nurses exclusively from tertiary hospitals; we did not recruit nurses from specialized hospitals or community health centers. Future studies should involve a wider spectrum of institutions in terms of type and level to determine whether the findings generalize. Cultural and institutional context: Management systems differ between countries and even between hospitals. Our findings may not be generalizable to other cultural contexts. Replication of this model in different contexts would test its cross‐cultural robustness.

## 8. Conclusion

Our study analyzed the correlation between the inclusive leadership, psychological safety, AI readiness, and the level of AI literacy among nurses. It also expanded on the concept of the role of leadership behaviors in terms of influencing technological skills indirectly through the work environment, psychological safety, and readiness to act. We are providing a new theory and practical application to our research, which will be used as a reference point in further studies and can provide useful information about how nursing managers and educators can encourage the development of AI capability. Therefore, it is necessary that the organizations and the management do not only promote the inclusiveness in leadership but also consider the implications of these actions on the psychological security and AI preparedness of nurses and ensure the practice of nursing is aligned with the needs of the AI healthcare and create an employee base that is highly literate in AI.

## Funding

This work was supported by the Henan Provincial Health Department Project (Grant number: LHGJ20250527) and the Henan Provincial Special Research Project for Building a Strong Education Province (Grant no. 2026JYQS073).

## Disclosure

All research was conducted independently to ensure the objectivity and impartiality of the study.

## Conflicts of Interest

The authors declare no conflicts of interest.

## Data Availability

The data that support the findings of this study are available on request from the corresponding authors. The data are not publicly available due to privacy or ethical restrictions. The authors affirm that the methods used in the data analyses are suitably applied to their data within their study design and context, and the statistical findings have been implemented and interpreted correctly. The authors agree to take responsibility for ensuring that the choice of statistical approach is appropriate and is conducted and interpreted correctly as a condition to submit to the Journal.
